# Comparing the order of the London Measure of Unplanned Pregnancy and the Demographic and Health Survey question on pregnancy intention in a single group of postnatal women in Malawi - the effect of question order on assessment of pregnancy intention

**DOI:** 10.1186/s13104-018-3577-1

**Published:** 2018-07-17

**Authors:** Jennifer A. Hall, Judith Stephenson, Geraldine Barrett

**Affiliations:** Research Department of Reproductive Health, UCL Institute for Women’s Health, London, UK

**Keywords:** Pregnancy intention, Measurement, London Measure of Unplanned Pregnancy, Demographic and Health Survey, Malawi

## Abstract

**Objective:**

To investigate the effect of question order on women’s responses to the London Measure of Unplanned Pregnancy (LMUP) or the pregnancy intention question of the Demographic and Health Survey (DHS) when both are asked in the same survey. We collected data on pregnancy intention from a cohort of 4244 pregnant women in Malawi who were re-interviewed at 1, 6 and 12 months postnatally. Women in Zone 1 were asked the LMUP, then antenatal questions, then the DHS pregnancy intention question, women in Zone 2 were asked the DHS pregnancy intention question, then antenatal questions, then the LMUP; women in Zone 3 were only asked the DHS pregnancy intention question. We used linear regression to compare the LMUP score and ordinal regression to compare DHS categorisations of pregnancy intention across Zones, adjusting for baseline socioeconomic differences between the Zones.

**Results:**

We found no effect of question order on the assessment of pregnancy intention by the LMUP. There were differences in the assessment of pregnancy intention when the pregnancy intention question in the DHS was used, however this seemed to be due to baseline sociodemographic differences between the groups of pregnant women being compared, and not due to question order.

**Electronic supplementary material:**

The online version of this article (10.1186/s13104-018-3577-1) contains supplementary material, which is available to authorized users.

## Introduction

Questions about pregnancy intention have been asked in large scale surveys around the world for over 50 years [1]. The purpose of these questions is to estimate the proportions of women with intended (or unintended) pregnancies and to use this information to understand the levels of desired fertility, need for family planning, and population growth patterns [2, 3]. Since the 1980s the main source of information on pregnancy intention in developing countries has been the Demographic and Health Surveys (DHS), based on a question asked up to 5 years after a birth: “At the time you became pregnant, did you want to become pregnant then, did you want to wait until later, or did you not want to have any (more) children at all?” The responses are categorized, respectively, as “intended”, “mistimed” and “unwanted” pregnancy, with “mistimed” and “unwanted” combined to estimate “unintended” pregnancy.

The DHS question follows a conceptualisation that was developed in the United States via the Growth of American Families Surveys in the 1950s [4], the National Fertility Surveys in the 1960s and 1970s [5, 6], and has continued from the 1970s to the present data with the periodic National Survey of Family Growth (NSFG) [7]. Over the last 20 years, however, there has been discussion about the validity of methods to measure pregnancy intention, particularly given the increased complexity of family formation patterns worldwide, the critiques of models of rational action within reproductive health, and the growing contribution of psychometric methods of measure development to all areas of social and health measurement [8–13]. As a response, the London Measure of Unplanned Pregnancy (LMUP) was developed in the early 2000s [14, 15]. It is a psychometrically valid and reliable tool comprising six questions which produce a score of 0–12, with higher scores indicating a more planned/intended pregnancy. The LMUP is now widely used, with eleven validated language versions across nine countries and more in progress [16–23]. Naturally, there has been a desire to compare the LMUP with other forms of measurement of pregnancy intention [24, 25], however we have been concerned about the best way to do this [26, 27] given the findings of Kaufmann et al. [28].

In the 1990s Kaufmann et al. carried out an experiment within the Arizona Women’s Health Survey. Using a randomized crossover design, they asked women two sets of pregnancy intention questions: the question sequence from the National Survey of Family Growth and a single question closely based on the DHS question. Women were randomised to which question they answered first, with the subsequent pregnancy intention question separated by a body of intervening items on sexual experience and contraceptive use. The findings showed that the NSFG and DHS questions yielded similar proportions of “intended”, “mistimed” and “unwanted” pregnancies, yet a quarter of women gave discordant responses and there was an effect of question order: “the percentage of pregnancies classified as mistimed was greater in response to whichever intendedness question was presented later” in the survey [28] (p. 814–5). This finding led Kaufmann et al. to question the validity of the underlying concepts, particularly “wantedness”. For us, it also leaves open the question of whether it is feasible to compare the LMUP with other pregnancy intentions questions simply by asking individual women two different sets of questions within one survey.

The fact that preceding questions, or the context of the survey, can affect how individuals respond to a particular survey question is well known, usually described as a ‘framing effect’. There have been various investigations into framing effects. Recent studies examining question order in surveys, on topics as diverse as opinions on assisted dying and reported experiences of bullying to rankings of priorities in a Delphi Survey, have found significant differences in responses according to where questions are placed [29–31]. A small body of work exists around single questions on general health status (e.g. Would you say your health in general is excellent, very good, good, fair, or poor?). These studies show that responses to the question on general health status vary according to whether the question is placed before or after other questions on health or life satisfaction, although the effects can vary in size and by language [32–36]. There has also been some examination of the effects of instrument order. For instance, experiments varying the order of general health-related quality of life measures with disease/condition-specific measures, which are often asked together in surveys, have generally shown little effect on either [37–40] or an effect only in some domains, such as mental health [41]. One study examined the effect of framing on a validated instrument, the Hospital Anxiety and Depression Scale (HADS), and found that preceding questions affected responses to the HADS [42].

In order to assess the effect of question order on women’s responses to the LMUP (a validated instrument) and the DHS question on pregnancy intention (a single survey question), we use data from a cohort study of pregnant women in Malawi [43].

## Main text

### Methodology

We collected data on pregnancy intention from a cohort of over 4200 pregnant women in Mchinji District, Malawi from March 2013 to July 2014. The methodology for recruiting and following up the cohort, and a description of the women included, have been described elsewhere [43]. Women were interviewed antenatally and at around 1-to-2, 6, and 12 months after the end of pregnancy. The LMUP was asked antenatally and at each postnatal follow up. The DHS question on pregnancy intention was only asked postnatally, as per standard practice.

Twenty-five clusters were randomly selected from 49 pre-defined areas covering the whole of Mchinji District [44]. These were grouped into three geographical Zones; 1, 2 and 3. To investigate any effect of question order, the LMUP and DHS questions were asked in a different order postnatally in each Zone. Women in Zone 1 were asked the LMUP first, followed by questions on antenatal issues, before being asked the DHS question. Women in Zone 2 were asked the DHS question, then the antenatal questions and then the LMUP. Finally, women in Zone 3 were only asked the DHS question.

**Fig. 1:**
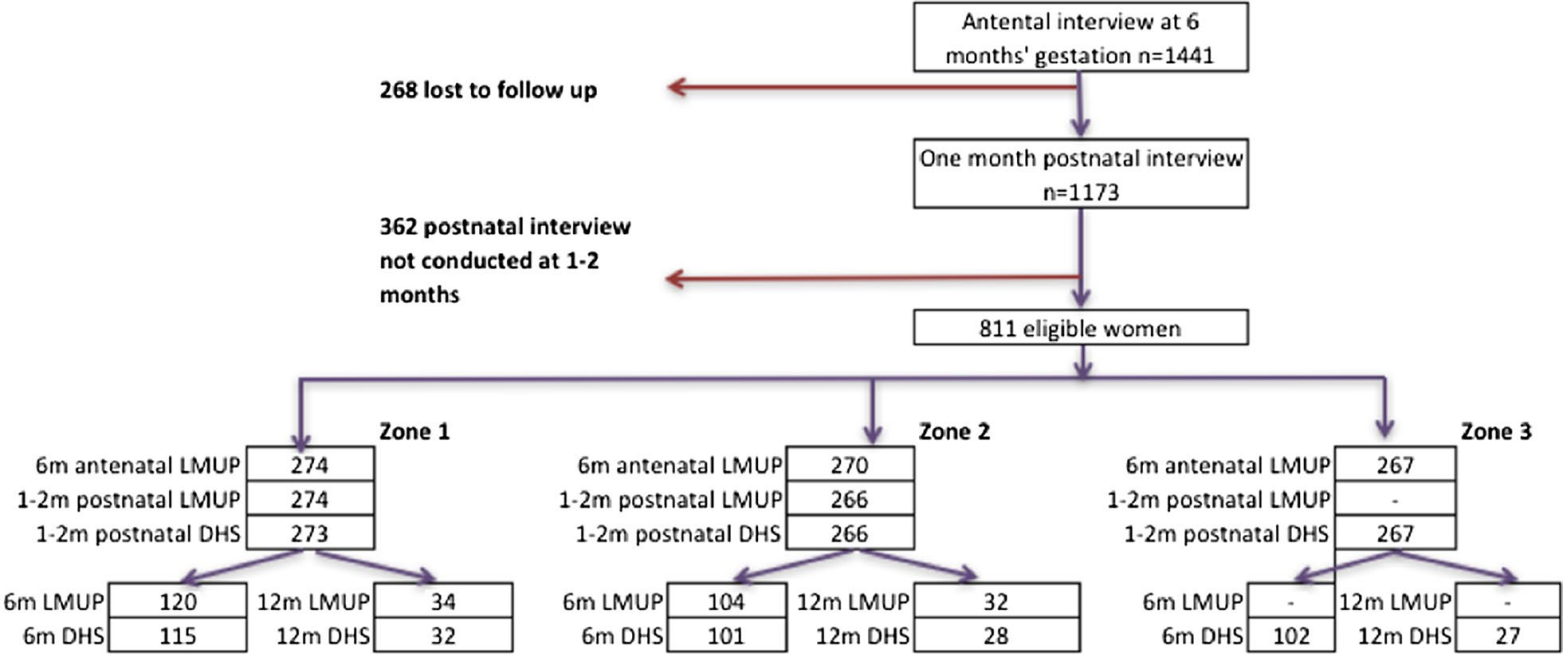
Flow chart of women who completed antenatal interview at 6 months’ gestation and each postnatal follow up

We examined the effect of question order by comparing the LMUP score (Zones 1 and 2 only) or DHS categorisation (all three Zones) at postnatal follow ups at 1-to-2 and 6 months. We did not include the 12-month data due to the small numbers at this time point (see Fig. 1). We compared the LMUP scores across the Zones using linear regression of the full zero to twelve score with robust standard errors, as recommended when using the LMUP as an outcome measure [45]. Power calculations confirmed that we had > 95% power to detect a difference of at least 0.3 points on the LMUP scale, a difference not deemed to be clinically significant. We used ordinal logistic regression to compare the DHS categorisations of intended, mistimed and unwanted across the Zones. We used the command “omodel” to test the proportional odds assumption and where this was violated we used the “gologit2” command to autofit a partial proportional odds model [46].

Given evidence of the determinants of pregnancy intention [43], we looked and adjusted for baseline differences in socio-economic status, marital status, maternal age, maternal education and number of live children between the Zones to ensure that we were only seeing the effect of question order. Variables were removed in a manual backwards stepwise fashion, starting with the largest p value and finishing when all variables with p-values > 0.1 had been removed. Zone remained in the model regardless of p-value as this was the variable of interest. To account for the fact that reported intention can change over time and to increase the comparability of the groups, we restricted the analysis to women who had been interviewed at 6 months antenatally and were interviewed postnatally at 1-to-2 and 6 months. All analyses were conducted in STATA version 15 (StataCorp. 2017. Stata Statistical Software: Release 15. College Station, TX: StataCorp LLC).

### Results

Figure 1 shows the number of women completing the LMUP and/or DHS at each postnatal follow up in each Zone. There were statistically significant differences between the Zones at baseline: socio-economic status (SES) (p < 0.001), education level (p = 0.006), age (p = 0.031), marital status (p = 0.018) and number of live children (p = 0.009) (see Additional file 1: Table S1).

#### LMUP

There were no significant differences between the LMUP scores in Zones 1 and 2 at either of the postnatal follow-ups, even without adjusting for the baseline differences in socio-demographics, as shown in Table 1. Multivariate models were created to check for negative confounding but Zone remained insignificant in these models at both time points. There was no significant difference in the proportion of women who changed their LMUP score between either antenatal and 1–2 month postnatal (p = 0.733) or between 1 and 2 months and 6 months postnatally (p = 0.941) suggesting that there was no effect of question order on the stability of the LMUP.

**Table 1:** Comparison of LMUP scores in Zones 1 and 2 at each postnatal visit

	Zone 1 LMUP then DHS	Zone 2 DHS then LMUP	Unadjusted p value	Adjusted p value
1–2 months postnatal				
n	274	266		
Mean (SD)	8.17 (4.10)	7.98 (3.29)	0.551	0.203^a^
Median (IQR)	10 (3–11)	9 (5–10)		
Six months postnatal				
n	120	104		
Mean (SD)	7.78 (4.34)	7.75 (3.57)	0.962	0.772^b^
Median (IQR)	10 (2–11)	10 (3–10)		

*SD* standard deviation, *IQR* interquartile range

^a^Adjusted for woman’s age, number of live children, marital status and education level

^b^Adjusted for woman’s age, number of live children and marital status

#### DHS

There was a statistically significant difference in the proportion of pregnancies categorised as intended on the DHS measurement of pregnancy intention across the Zones at the first postnatal visit (p = 0.025), as shown in Table 2. Once baseline differences in socio-demographics between the Zones were controlled for, the differences in the DHS categorisations were not statistically significant (p = 0.177). For the analysis at 6 months postnatally, a partial proportional odds model had to be fitted for the univariate model as the assumption of proportional odds was violated. There was a borderline significant difference between the Zones (p = 0.087) which again became non-significant when adjusted for socio-demographics on multiple ordinal regression (p = 0.992). There was no significant difference in the proportion of women who changed their DHS categorisation between 1 and 2 months and 6 months postnatally (p = 0.488) suggesting that there was no effect of question order on the stability of the DHS.

**Table 2:** Comparison of DHS categorisations in Zones 1, 2 and 3 at each postnatal visit

	Zone 1 LMUP then DHS	Zone 2 DHS then LMUP	Zone 3 DHS only	Unadjusted p value	Adjusted p value
DHS postnatal 1–2 months					
Intended n (%)	166 (61)	189 (71)	148 (55)		
Mistimed n (%)	66 (24)	26 (10)	80 (30)	0.025	0.177^a^
Unwanted n (%)	41 (15)	51 (19)	39 (15)		
Total	273	266	267		
DHS postnatal 6 months					
Intended n (%)	73 (63)	69 (68)	63 (62)		
Mistimed n (%)	20 (17)	9 (9)	29 (28)	0.087^b^	0.992^c^
Unwanted n (%)	22 (19)	23 (23)	10 (10)		
Total	115	101	102		

^a^Adjusted for woman’s age, number of live children and marital status

^b^Partial proportional odds ordinal logistic regression

^c^Adjusted for woman’s age, number of live children and marital status

### Conclusions

We found no effect of question order on the LMUP score at either postnatal time point, in either unadjusted or adjusted analyses. We found no effect of question order on the DHS categorisations at either postnatal time point once we had adjusted for baseline socio-demographic factors. We therefore conclude that there was no effect of question order on either measure of pregnancy intention.

Kaufmann et al. found evidence of an effect on question order in their data, in particular they found more “mistimed” pregnancies in response to whichever question was asked second [28]. In our data, had we just compared Zone 1 (LMUP then DHS) with Zone 2 (DHS then LMUP) then our findings would be the same as Kaufmann et al. This is because there was a higher proportion of “mistimed” pregnancies in Zone 1, where the DHS was asked second, than there was in Zone 2, where the DHS was asked first, at both postnatal time points. However, Zone 3, where only the DHS was asked, had the highest proportion of “mistimed” pregnancies at both postnatal time points. Since women in Zone 3 were not asked the LMUP postnatally, the proportion of mistimed pregnancies could not have been influenced by the LMUP. This suggests that the differences in the proportion of mistimed pregnancies between the Zones were not due to question order. Indeed, when baseline socio-demographic differences were accounted for, the differences in DHS categorisation across the Zones were no longer significant. In contrast, despite the differences in baseline socio-demographic factors across the Zones, the LMUP was not significantly different between the Zones at any time point, indicating that this more nuanced measure of pregnancy intention is probably more reliable than the DHS.

The lack of an effect of question order in our analyses is encouraging as it suggests that it is possible to compare measures of pregnancy intention by means of comparisons within individuals in the context of a survey.

## Limitations

We were not able to randomise question order at the individual level, meaning that known and unknown confounders were not balanced across the Zones. However, we were able to adjust for known confounders. We can only conclude that there is no effect of question order on reported pregnancy in the Chichewa language; others have found that the effect of question order may differ by language, so our finding should be verified in other languages [34].

## Additional file

**Additional file 1: Table S1.** Baseline characteristics of the women by Zone. A table comparing the sociodemographic characteristics of the women in each Zone at baseline.

## Abbreviations

DHS: Demographic and Health Survey
LMUP: London Measure of Unplanned Pregnancy
NSFG: National Survey of Family Growth
