# Supplementary material for: Comparing the order of the London Measure of Unplanned Pregnancy and the Demographic and Health Survey question on pregnancy intention in a single group of postnatal women in Malawi - the effect of question order on assessment of pregnancy intention
Source: BMC Res Notes. 2018 Jul 17;11:487. doi: 10.1186/s13104-018-3577-1 (PMC6050738; doi:10.1186/s13104-018-3577-1)
Supplement: Supplementary file 1 — Additional file 1: Table S1. Baseline characteristics of the women by Zone. A table comparing the sociodemographic characteristics of the women in each Zone at baseline. [file 13104_2018_3577_MOESM1_ESM.docx]

Additional file 1 Baseline characteristics of the women by Zone

|  |  | **Zone** | | | | | |  |
| --- | --- | --- | --- | --- | --- | --- | --- | --- |
|  |  | **1** | | **2** | | **3** | | **p value** |
|  |  | **n** | **%** | **n** | **%** | **n** | **%** |  |
| SES status quintile | Poorest | 27 | 10.1 | 63 | 23.5 | 61 | 23.2 | <0.001 |
|  | Second | 57 | 21.3 | 48 | 17.9 | 61 | 23.2 |  |
|  | Middle | 60 | 22.4 | 66 | 24.6 | 57 | 21.7 |  |
|  | Next-rich | 54 | 20.2 | 58 | 21.6 | 50 | 19.0 |  |
|  | Richest | 70 | 26.1 | 33 | 12.3 | 34 | 12.9 |  |
| Mother's education level | None | 23 | 8.4 | 40 | 14.8 | 26 | 9.7 | 0.006 |
|  | Primary | 195 | 71.2 | 197 | 73.0 | 212 | 79.4 |  |
|  | Secondary | 55 | 20.1 | 33 | 12.2 | 29 | 10.9 |  |
|  | Tertiary | 1 | 0.4 | 0 | 0.0 | 0 | 0.0 |  |
| Marital status | Married | 245 | 89.4 | 258 | 95.6 | 250 | 93.6 | 0.018 |
|  | Unmarried | 29 | 10.6 | 12 | 4.4 | 17 | 6.4 |  |
| Mother's age group | 15-17 | 27 | 9.9 | 28 | 10.4 | 16 | 6.0 | 0.031 |
|  | 18-29 | 190 | 69.3 | 170 | 63.0 | 167 | 62.6 |  |
|  | 30 and over | 57 | 20.8 | 72 | 26.7 | 84 | 31.5 |  |
| Number of live children | None | 94 | 34.3 | 88 | 32.6 | 77 | 28.8 | 0.009 |
|  | One to three | 142 | 51.8 | 132 | 48.9 | 116 | 43.4 |  |
|  | Four or more | 38 | 13.9 | 50 | 18.5 | 74 | 27.7 |  |
| Notes: n=274 for Zone 1, 270 for Zone 2 and 267 for Zone 3, except for SES where n=268, n=268 and n=263 respectively due to missing data. SES: socio-economic status. | | | | | | | | |
